# Role of maize kernels on the fitness of *Spodoptera frugiperda* (Lepidoptera: Noctuidae)

**DOI:** 10.1093/jisesa/ieag104

**Published:** 2026-09-26

**Authors:** Seyedeh Masoumeh Hosseini Mousavi, Seyed Ali Hemmati, Arash Rasekh

**Affiliations:** Department of Plant Protection, Faculty of Agriculture, Shahid Chamran University of Ahvaz, Ahvaz, Iran; Department of Plant Protection, Faculty of Agriculture, Shahid Chamran University of Ahvaz, Ahvaz, Iran; Department of Plant Protection, Faculty of Agriculture, Shahid Chamran University of Ahvaz, Ahvaz, Iran

**Keywords:** noctuid species, feeding performance, enzymes activities, two-sex life table, integrated pest management

## Abstract

Recent outbreak of the fall armyworm, *Spodoptera frugiperda* (JE Smith), has caused considerable challenges to maize production, *Zea mays* L., in the world. Sustainable pest management through host plant resistance requires further confirmation of maize hybrids that reduce pest pressure during postflowering stage. In this study, the comparative resistance of kernels from 10 maize hybrids, including BC678, SC201, SC704, SC410, SC706, SC400, SC260, BK66, SC705, and BK65, was evaluated using the nutritional indices, demographic parameters, and enzymatic activities of *Spodoptera frugiperda* in relation to kernel biochemical traits. The selected hybrids are widely cultivated in Iran and represent the major maturity classes. Larvae fed on BC678 and SC705 exhibited lower efficiencies of conversion of ingested (ECI) and digested food (ECD), accompanied by higher relative metabolic rate and metabolic cost. In contrast, higher ECI and ECD values were observed on SC704 and SC400. The intrinsic rate of increase (*r*) ranged from 0.110 to 0.169 day^−1^ across treatments, with the lowest value recorded on SC705 and higher values on SC410 and SC706. Lower finite rate of increase (*λ*) and gross reproduction rate were also obtained on SC705. Decreased amylase activity was recorded on SC705. Moreover, larvae reared on SC705 displayed the highest peroxidase activity, while those reared on BC678 and SC705 showed increased superoxide dismutase activity. Variations in the concentrations of primary and secondary metabolites in the kernels significantly affected pest performance. The clustering results additionally classified SC705 as the least suitable hybrid for *S. frugiperda*, indicating its potential as a source of host plant resistance for maize breeding and as a component of integrated pest management programs to reduce insect damage during kernel development.

## Introduction

Maize, *Zea mays* L. (Poaceae), is a globally important cereal crop that is widely used as a human food, livestock feed, and natural raw material for starch and starch-based industrial products (Amanjyoti et al. 2024, Swati et al. 2024, Tajdar et al. 2025). Kernels are the important and edible part of the maize plant, containing starch, essential vitamins, selenium, potassium, and bioactive compounds, and their germ is a rich source of oil, protein, and soluble and insoluble materials (Eckhoff et al. 2003, Kumar and Jhariya 2013, Rouf Shah et al. 2016). The leaves and stems, other parts of the plant, are processed into silage and used as winter animal feed (Klopfenstein et al. 2013). Maize production in many Asian countries suffers yield losses ranging from 15% to 73% due to insect pests, particularly after the recent expansion of the fall armyworm, *Spodoptera frugiperda* (JE Smith) (Lepidoptera: Noctuidae) populations (Shylesha et al. 2018, Wu et al. 2022, Qi et al. 2024, Yan et al. 2025). Since the initial report of *S. frugiperda* in Iran in 2023 (Naseri et al. 2024), ongoing field surveillance has recently confirmed its expansion into maize fields in Khuzestan province. This growing threat is likely fueled by drought and fluctuating climatic conditions, combined with the pest’s high migration capacity, lack of diapause, short generation time, and high reproductive potential, all of which facilitate its rapid establishment and population growth (Chen et al. 2020, Gebretsadik et al. 2023).

*Spodoptera frugiperda* attacks more than 353 plant species belonging to over 76 botanical families and causes substantial economic damage, with maize as the most seriously impacted crop (Montezano et al. 2018). Early instars mainly feed on leaves, especially the whorl leaves in maize, though stem feeding may occur under heavy infestations (Gilal et al. 2022). Larval feeding on maize ears by later instars has also been reported during the postflowering period, which adversely affects kernel weight, kernel density, and total yield (Ni et al. 2024), with reported maize grain yield losses of approximately 22% under field conditions (Yaméogo et al. 2024). Insecticides are the primary management tool used by farmers against *S. frugiperda* and their usage has recently enhanced in attacked areas (Yang et al. 2021). However, the widespread use of insecticides promotes pesticide resistance, weakens the effectiveness of natural enemies, creates serious health and environmental risks, and causes economic problems (Castle 1999, Schmidt-Jeffris et al. 2021). The cultivation of resistant maize hybrids is an environmentally friendly strategy that can decrease the need for insecticide applications and their associated environmental risks (Jafari et al. 2025b).

Plants have evolved complex defense strategies against insect pests, with secondary metabolites playing a key role in protecting them from herbivory (War et al. 2012, Alekaram et al. 2024, Jafari et al. 2025b). The most prominent secondary metabolites in maize plants are flavonoids, anthocyanins, phenolic acids, tannins, protease inhibitors, and chlorogenic acid, which serve as both constitutive and inducible defense mechanisms, and thus, provide plants with a multilayered chemical resistance strategy (de Paiva et al. 2016, Jafari et al. 2025b). Flavonoids possess antifeedant activity and play important roles in plant defense by modulating insect feeding behavior, growth, development, and oviposition (Treutter 2006, Jackowski et al. 2017, Chatterjee et al. 2023). Anthocyanins are typically associated with stress responses and may contribute in antibiosis resistance by interfering with insect nutritional physiology (Jafari et al. 2025b). Phenolic compounds produce oxidation metabolites that attach to leaf proteins and interfere with protein digestion in insects (Bhonwong et al. 2009, War et al. 2012). Other biochemicals also affect insect pests through antinutritive, antidigestive, or toxic effects (War et al. 2012). The level of primary metabolites, including carbohydrates and proteins, may also be involved in regulating insects’ populations because their body size and, consequently, life history traits are closely associated with nutrient availability (Awmack and Leather 2002, Arab Yabarati et al. 2025).

Identifying and using pest-resistant maize hybrids with potent chemical defenses could support the development of sustainable management programs (Mihm et al. 1988). Chiriboga Morales et al. (2021) assessed the resistance of 6 African maize cultivars to *S. frugiperda* and found that SC Duma 43 conferred resistance primarily through antibiosis. Zhang et al. (2023) studied the population performance of *S. frugiperda* on several maize varieties and reported that Zhengdan 958 was a nonpreferred host due to its suppressive effects on pest population growth. Assessing the resistance of 10 maize hybrids to *Leucania loreyi* (Duponchel), Jafari et al. (2025b) reported that the foliar profiles of either primary or secondary metabolites in hybrids SC706 and SC260 were responsible for impaired insect performance.

Insect performance on host plants, as documented in various studies, is frequently quantified by various biological and physiological traits (Hosseini Mousavi et al. 2023, Alekaram et al. 2024, Jafari et al. 2025a, 2025b). Nutritional indices are widely used to evaluate the efficiency of food consumption, digestion, and conversion into biomass, thereby providing important information on host plant suitability (Arab Yabarati et al. 2025). Likewise, life history and life table parameters offer comprehensive insights into the effects of host plants on insect survival, development, reproduction, and population growth. They are valuable tools for evaluating host plant resistance and predicting the potential population growth of insect pests on different crop genotypes (Zhang et al. 2023, Jafari et al. 2025b). The insects’ digestive and antioxidant enzymes also play essential roles in nutrient utilization and reflect the insect’s capacity to cope with defensive compounds and nutritional stress (Jafari et al. 2025b). Among the digestive enzymes, proteases facilitate protein digestion by hydrolyzing dietary proteins into absorbable peptides and amino acids, whereas amylases convert starches and complex polysaccharides into simpler sugars like maltose and glucose (Terra et al. 1996, Franco et al. 2002). Insect antioxidant enzymes, including superoxide dismutase (SOD), peroxidase (POD), and catalase (CAT), are also upregulated as part of their defense against increased production of reactive oxygen species (ROS) (Łukasik et al. 2011, Deska 2020). This response is often induced by the pro-oxidant secondary metabolites found in plants, which can generate various ROS such as H_2_O_2_, O^−2^, and OH^−^ (Livingstone 2001). Excessive ROS can damage vital cellular components like proteins, lipids, and DNA, which consequently weakens insect growth (Kodrík et al. 2015).

Screening programs for identifying the maize hybrids resistant to *S. frugiperda* or other noctuid species have been widely implemented (Alekaram et al. 2024, Ni et al. 2024, Jafari et al. 2025a, 2025b). However, focusing on the effects of feeding on different plant structures of maize hybrids to this newly established pest is still limited (Gilal et al. 2022). Using the 10 different maize hybrids, the objectives of this study were to assess the kernel-mediated resistance of the experimental hybrids to *S. frugiperda* by assessing the insect nutritional indices, demographic traits, and enzymatic activities following feeding on the kernels. We also measured primary and secondary metabolite levels in maize hybrids kernels as a possible explanation of any variation observed in *S. frugiperda* performance. Although some of these maize hybrids have been evaluated against other lepidopteran pests (Alekaram et al. 2024, Jafari et al. 2025a, 2025b), their resistance to *S. frugiperda* remains unknown. The findings can be important for identifying germplasm with resistance to ear feeding and can provide growers with a more practical and cost-effective strategy to restrict *S. frugiperda* populations on ears before they cause considerable yield loss.

## Materials and Methods

### Collection and Rearing of *Spodoptera frugiperda*

A stock colony of *S. frugiperda* was founded using larvae obtained from naturally infested maize fields in Dezful (Khuzestan Province, Iran) during the 2025 growing season. The larvae were brought to the laboratory and maintained separately on each of the 10 maize hybrids evaluated in this study (see the Maize hybrids subsection). They were fed on harvested fresh maize ear pieces containing kernels and cob. Upon adult emergence, moths were introduced into cylindrical rearing cages (35 × 25 cm), supplied with a potted maize plant of the respective hybrid at the 4-leaf stage for oviposition. Each pot (10 cm diam) contained field soil. Adult nutrition was provided through cotton balls soaked in 10% honey solution. Egg masses deposited on the plants were removed every day and incubated until hatching to obtain the F1 larvae. Second-generation (F2) eggs or larvae, including third and final instars obtained from synchronized egg cohorts, were selected for further experiments. The experimental unit for all demographic and nutritional indices studies was a single larva. Each larva was individually reared in a separate container (10-cm Petri dishes) and randomly assigned to 1 maize hybrid, ensuring that observations represented independent biological replicates. Insects were reared and tested in a growth chamber at 25 ± 1 °C, 65 ± 5% relative humidity, and 16:8 light:dark photoperiod.

### Maize Hybrids

Seeds of 10 commercial maize hybrids, including SC201, SC260 (early maturing), SC400, SC410, BK65, BK66 (medium maturing), BC678, SC704, SC706, and SC705 (late maturing) were obtained from the Seed and Plant Improvement Research Institute (Karaj, Iran) and sown in a research field in Ahvaz, Khuzestan province, southwestern Iran. These hybrids were selected based on their widespread cultivation in Iran and their representation of the major maturity classes adapted to different climatic and production conditions across the country. As detailed information on their genetic background and documented resistance traits is not publicly available from the breeder, previous reports of variation in *L. loreyi* performance among these hybrids (Jafari et al. 2025a, 2025b) were also considered to ensure representation of a broad range of host responses. The seeds were cultivated on raised beds (15 cm high and 30 m long) using 70-cm row spacing and 15 cm plant spacing. The study field was arranged into 20 rows, with each maize hybrid represented by 2 rows. Agronomic operations were performed following standard practices of the region. During the entire growing season, foliar applications of insecticides were avoided, and any insects or egg masses found on plants were removed by hand. When the plants entered the reproductive phase, ears were harvested for subsequent larvae feeding.

### Nutritional Indices of *Spodoptera frugiperda*

To determine the nutritional indices of *S. frugiperda* on the examined maize hybrids, 25 third instars per treatment were individually weighed and placed in 10-cm Petri dishes. Fresh dehusked maize ears were previously cut into small sections containing kernels attached to the cob and provided to the larvae. The larvae were permitted to feed on preweighed fresh maize ear pieces of each maize hybrid for 24 h. After each feeding period, larval weight gain along with the weight of leftover food and frass from the insects were measured with the help of a digital scale (Sartorius AG, Germany; model GCA803S). Larval food was replenished with new fresh pieces of maize ears every day until the larvae pupated. Prepupal and pupal weights were also measured individually. Larval, food, and frass dry weights were measured after oven-drying (UN110, Memmert) the samples at 60 °C for 48 h. These dry matter values were used to compute the nutritional indices as reported by Waldbauer (1968) and Scriber and Slansky (1981). The efficiency of conversion of ingested food (ECI), efficiency of conversion of digested food (ECD), and relative growth rate (RGR) were estimated as follows:


$$\begin{array}{c} E\mathrm{CI}=\frac{\;P}{E}\times 100\\ \begin{array}{c} E\mathrm{CD}=\frac{\;P}{E-F\;}\times 100\\ R\mathrm{GR}=\frac{P}{W_{0}\times T} \end{array} \end{array}$$

Other indices, including consumption index (CI) = *E*/*A*, approximate digestibility (AD) = (*E*−*F*)/*E* × 100, relative consumption rate (RCR) = *E*/(*W*_0_×*T*), relative metabolic rate (RMR) = *M*/(*W*_0_×*T*), and metabolic cost (MC) = 100 − ECD were also calculated, where *P* is the dry weight gain by larvae (mg), *W*_0_ is the primary weight of larvae (mg), *T* is the feeding period (days), *E* is the dry weight of food consumed (mg), *F* is the dry weight of frass produced (mg), *A* is the mean dry weight of insect during feeding period (mg), and *M* = (*E*−*F*)−*P* = food metabolized (mg).

### Demographic Parameters of *Spodoptera frugiperda*

Eggs masses (*n *= 100, F2 generation) of *S. frugiperda*, deposited within 24 h on each hybrid, were monitored for each maize hybrid in a completely randomized design. Eggs with their leaf substrate transferred to 10-cm Petri dishes with moistened filter paper and ventilated lids and initially maintained together until hatching. Egg duration and survival were assessed daily. Immediately after hatching, each neonate was individually transferred into new Petri dishes (1 larva per dish) and fed daily with fresh pieces of dehusked maize ears of the respective maize hybrids until pupation. The food was provided as needed. The life history parameters obtained from daily observations were egg period, egg survival, larval period, larval survival, prepupal period, prepupal survival, pupal period, pupal survival, and sex ratio.

Following adult emergence, 2 newly emerged pairs reared on the same maize hybrid were transferred into an oviposition cage, containing a potted plant of the corresponding maize hybrid (described in the section of the origin and rearing of *S. frugiperda*). This was repeated for all adults on different treatments. A solution of honey (10%) was provided as food source. Moths were moved daily to clean cages, and eggs laid in the previous cages were counted. Accordingly, other life history parameters, including adult longevity, fecundity (eggs/female), adult preoviposition period (APOP) (time from eclosion to first oviposition), oviposition period, and total preoviposition period (TPOP) (time from egg hatching to first oviposition) of *S. frugiperda* were assessed on each maize hybrid until all moths died.

### Digestive Enzymes of *Spodoptera frugiperda*

Twenty last instar larvae of *S. frugiperda* were randomly selected from the same maize hybrid on which they had been previously reared. The larvae were chilled, dissected under a stereomicroscope (Stemi SV6 ZEISS, Germany), and their midguts were homogenized with a handheld glass homogenizer in distilled water. The homogenates were subsequently centrifuged at 12,000 × *g* for 10 min. The collected supernatants were stored in screw-cap microtubes (1.5 mL) at −20 °C for subsequent assays.

The proteolytic activity was quantified following the method reported by Elpidina et al. (2001). Initially, a reaction mixture with 80 μL azocasein (1.5%), 50 mM universal buffer (sodium phosphate-borate, pH = 11), and 50-μL insect extracts was held at 37 °C for 50 min for incubation. The reaction was ended by 100 μL trichloroacetic acid (30%) at 4 °C for 30 min and centrifuged at 16,000 × *g* for 10 min. Following the addition of 2 M NaOH, absorbance was taken at 440 nm.

The amylolytic activity assay was carried out according to Bernfeld (1955) methodology with 1% starch as the substrate and dinitrosalicylic acid as the colorimetric agent. The larvae extract (50 μL) was incubated with 20 μL starch (1%) and 250 μL universal buffer (50 mM succinate-glycine-2, morpholinoethan sulfunic acid, pH = 10) for 10 min, followed by mixing with 50 μL 3,5-dinitrosalicylic acid reagent. After heating (85 to 95 °C for 10 min) the mixture and centrifugation (16,000 × *g*, 4 °C, 5 min), absorbance was quantified at 540 nm. Each assay for enzyme activity was performed in three replicates, using corresponding blanks that contained no enzyme extract. A spectrophotometer was employed for all measurements (S2100SUV, UNICO, Tucson, Arizona, United States).

### Antioxidant Enzymes of *Spodoptera frugiperda*

Final instars (0.5 g) were randomly selected from the same maize hybrid on which they had been previously fed. The larvae were homogenized in potassium phosphate buffer (KH_2_PO_4_ and K_2_HPO_4_, 0.1 M, pH = 7.4) on ice, centrifuged at 5,000 × *g* for 15 min, and the resulting supernatants stored at −20 °C for subsequent assays (Sezer and Ozalp 2015).

The POD activity assay was carried out following van der Poll et al. (1994) methodology. The reaction mixture consisted of 50 µL enzyme extract, 1,000 μL potassium phosphate buffer (100 mM, pH = 7), 1,000 μL guaiacol (20 mM), and 1,000 µL hydrogen peroxide (H_2_O_2_) (10 mM). The absorbance changes due to guaiacol oxidation were measured at 470 nm over a 120-s reaction time.

SOD activity was assessed according to the procedure outlined by Beyer and Fridovich (1987). The reaction mixture included 400 μL enzyme extract, 1,000 μL sodium carbonate (200 mM), 100 μL nitro blue tetrazolium (NBT) (5 mM), 100 μL potassium phosphate buffer) 0.1 M, pH = 7.4), 200 μL methionine (0.25 M), and 150 μL riboflavin (0.1 mM). After incubation of mixture in dark for 30 min, the NBT reduction was determined at 560 nm.

CAT activity was determined following the procedure of Beers and Sizer (1952). First, 10 µL of insect extract was combined with 100 μL potassium phosphate buffer (0.1 M, pH = 7.4) and 50 µL H_2_O_2_ (3%). Then, the absorbance change due to H_2_O_2_ degradation was quantified at 240 nm with a microplate reader (INNO, LTEK, South Korea) during the 120-s reaction time. All enzymatic assays were conducted in 3 replicates with corresponding blanks contained no enzyme extract. A spectrophotometer was used for measurements of POD and SOD activities (S2100SUV, UNICO, Tucson, Arizona, United States).

### Determination of Primary and Secondary Metabolites in Maize Hybrids Kernels

To measure the primary (carbohydrate, starch, and protein) and secondary (phenol, flavonoid, and anthocyanin) metabolites contents in kernels, a completely randomized design assay was applied using 3 replicates for each analysis.

Carbohydrate content was estimated using the phenol–sulfuric acid method, which relies on photometric measurement of the color complex generated after reaction of the sample with sulfuric acid and a phenol solution (Lau et al. 2022).

The starch content was measured based on the method of Bernfeld (1955). Briefly, powdered kernels (200 mg) were homogenized in distilled water (35 mL) and heated to boiling. The homogenate (100 μL) was mixed with 2.5 mL iodine reagent (0.2% KI and 0.02% I_2_) and analyzed at 580 nm.

Protein concentration was computed by Kjeldahl’s (Kjeldahl 1883) method. Samples were digested with concentrated sulfuric acid (H_2_SO_4_) (25 mL) and a catalyst, distilled with 40% sodium hydroxide (NaOH) to release ammonia gas (NH_3_), and the liberated ammonia was trapped in 4% boric acid (H_3_BO_3_) containing methyl red indicator. Protein content was calculated from total nitrogen with a conversion coefficient of 5.25.

The phenol content was measured according to the method of Slinkard and Singleton (1977). Kernels (2 g) were ground, homogenized in 80% methanol, and centrifuged at 12,000 × *g* for 15 min. After addition of 1.5 mL Folin-Ciocalteu reagent (1:10 v v^−1^) to 0.1 mL of each extract, the solution was incubated for 30 min with 1.4 mL sodium carbonate (Na_2_CO_3_) (1 M). Absorbance readings were taken at 765 nm.

Flavonoid concentration was measured following Kim et al.’s  (2003) procedure. Briefly, kernels (2 g) were thoroughly homogenized in 3 mL acidified ethanol (99% ethanol containing 1% acetic acid) following grinding, and subsequently centrifuged at 12,000 × *g* for 15 min. The resulting supernatant was filtered through Whatman No. 1 filter paper and incubated in water bath at 80 °C for 5 min, following absorbance measurement at 270, 300, and 330 nm wavelength.

The determination of anthocyanin content followed the protocol of Kim et al.  (2003) described for flavonoid content, with a minor modification that acidified methanol (1% acetic acid in 99% methanol) was used for extraction and measurements was taken at 550 nm wavelength. All absorbance readings were recorded with a spectrophotometer (S2100SUV, UNICO, Tucson, Arizona, United States).

### Statistical Analysis

The data on nutritional indices and enzyme activities of the insect, together with the primary and secondary metabolic products of maize hybrids kernels were subjected to 1-way analysis of variance in SPSS version 16.0 after assessment of data normality and variance homogeneity with the Shapiro–Wilk and Levene’s tests, respectively. Mean comparisons were performed using Tukey’s HSD test (*P *< 0.05).

The life history data of *S. frugiperda* were processed using TWOSEX–MSChart software (Chi 2022), a computer package for conducting age-stage, 2-sex life table analysis (Chi and Liu 1985, Chi 1988) and deriving life table parameters. The intrinsic rate of increase (*r*), a life table parameter, was estimated using the binary iteration method from the Euler–Lotka formula with age (*x*) indexed from 0 (Huang and Chi 2013, Chi et al. 2023):


$$\sum_{x\;=\;0}^{\infty }\;e^{-r(x+1)}l_{x}m_{x}=1$$

where *l_x_* (age-specific survival) is the probability that an egg survives from oviposition to age *x* and *m_x_* (age-specific fecundity) is the mean number of daughters produced by each female at age *x*. Other life table parameters, including net reproduction rate $R_{0}=\sum_{x=0}^{\infty }{l_{x}m}_{x}$, gross reproduction rate $\mathrm{GRR}\;=\sum_{x=0}^{\infty }m_{x}$, finite rate of increase $\lambda =e^{r}$, and mean generation time $T=\ln R_{0}/r$ were also estimated (Carey 1993). Bootstrap resampling (100,000 random replications) was used to minimize variations and paired bootstrap test (*P *< 0.05) was used for comparison of means (Efron and Tibshirani 1993, Huang et al. 2018).

Pearson’s correlation was used to examine associations among the nutritional indices, demographic parameters, and enzyme activities of *S. frugiperda*, and the metabolic products of maize hybrids kernels. Cluster analysis was conducted in SPSS version 16.0 using the minimum-variance hierarchical clustering procedure proposed by Ward (1963), based on the nutritional indices, demographic parameters, and enzymatic activities of *S. frugiperda* on the tested treatments.

## Results

### Nutritional Indices of *Spodoptera frugiperda*

Rearing on the kernels of the examined maize hybrids significantly affected the nutritional indices of *S. frugiperda*, which are presented in Table 1. The ECI food (*F *= 19.60; df = 9, 240; *P *< 0.001) and ECD food (*F *= 23.48; df = 9, 240; *P *< 0.001) were lower on BC678 and SC705. However, higher associated values of ECI were recorded on SC704 and SC400, and those of ECD were obtained on SC704, SC400, and BK65. Larvae reared on BC678 and SC260 showed reduced values of RGR (*F *= 4.95; df = 9, 240; *P *< 0.001), whereas they exhibited the greatest RGR on SC410. The highest and lowest amounts of CI (*F *= 38.60; df = 9, 240; *P *< 0.001), RCR (*F *= 38.60; df = 9, 240; *P *< 0.001), and RMR (*F *= 44.41; df = 9, 240; *P *< 0.001) were obtained on SC705 and SC704, respectively. Higher values of AD (*F *= 52.90; df = 9, 240; *P *< 0.001) were obtained on SC706, SC260, and SC705, while the lowest amount of this index was on SC704. The MC (*F *= 23.48; df = 9, 240; *P *< 0.001) of larvae increased on BC678 and SC705, but declined on SC704, SC400, and BK65.

**Table 1.** Nutritional indices (mean ± SE) of *Spodoptera frugiperda* larvae fed on the kernels of 10 maize hybrids

Hybrids	ECI (%)	ECD (%)	RGR (mg mg^−1^ day^−1^)	CI (mg mg^−1^)	AD (%)	RCR(mg mg^−1^ day^−1^)	RMR (mg mg^−1^ day^−1^)	MC (%)
**BC678**	5.9 ± 0.3d	6.5 ± 0.4d	0.19 ± 0.01c	12.8 ± 0.5ab	91.8 ± 0.4bc	3.2 ± 0.1ab	2.7 ± 0.1ab	93.4 ± 0.4a
**SC201**	8.8 ± 0.4bc	9.4 ± 0.4bc	0.20 ± 0.00bc	9.5 ± 0.3c	94.1 ± 0.2ab	2.4 ± 0.1c	2.0 ± 0.1c	90.6 ± 0.4bc
**SC704**	11.3 ± 0.5a	13.7 ± 0.7a	0.21 ± 0.01abc	7.6 ± 0.2d	82.9 ± 0.8e	1.9 ± 0.1d	1.3 ± 0.1d	86.3 ± 0.7d
**SC410**	10.5 ± 0.4ab	11.6 ± 0.5ab	0.24 ± 0.01a	9.3 ± 0.2c	90.0 ± 0. 7c	2.3 ± 0.1c	1.8 ± 0.1c	88.3 ± 0.5 cd
**SC706**	7.9 ± 0.3 cd	8.3 ± 0.3 cd	0.22 ± 0.01ab	11.6 ± 0.3 b	95.3 ± 0.3a	2.9 ± 0.1 b	2.5 ± 0.1 b	91.7 ± 0.3ab
**SC400**	11.1 ± 0.7a	12.3 ± 0.8a	0.22 ± 0.01ab	8.4 ± 0.3 cd	90.1 ± 0.8c	2.1 ± 0.1 cd	1.7 ± 0.1 cd	87.6 ± 0.8d
**SC260**	8.6 ± 0.4bc	9.1 ± 0.5c	0.18 ± 0.01c	8.9 ± 0.3 cd	95.3 ± 0.3a	2.2 ± 0.1 cd	1.9 ± 0.1c	90.9 ± 0.5 b
**BK66**	7.0 ± 0.3 cd	7.6 ± 0.4 cd	0.20 ± 0.01bc	11.7 ± 0.4 b	93.2 ± 0.4ab	2.9 ± 0.1 b	2.5 ± 0.1 b	92.4 ± 0.4ab
**SC705**	6.2 ± 0.2d	6.6 ± 0.2d	0.21 ± 0.01abc	13.9 ± 0.4a	95.5 ± 0.3a	3.5 ± 0.1a	3.1 ± 0.1a	93.4 ± 0.2a
**BK65**	10.2 ± 0.5ab	11.9 ± 0.7a	0.21 ± 0.01abc	8.8 ± 0.2 cd	86.5 ± 0.9d	2.2 ± 0.1 cd	1.7 ± 0.1 cd	88.0 ± 0.7d

Means followed by different letters in each column are significantly different (Tukey’s HSD test, *P *< 0.05).

Abbreviations: AD, approximate digestibility; CI, consumption index; ECD, efficiency of conversion of digested food; ECI, efficiency of conversion of ingested food; MC, metabolic cost; RCR, relative consumption rate; RGR, relative growth rate; RMR, relative metabolic rate.

**Fig. 1.**
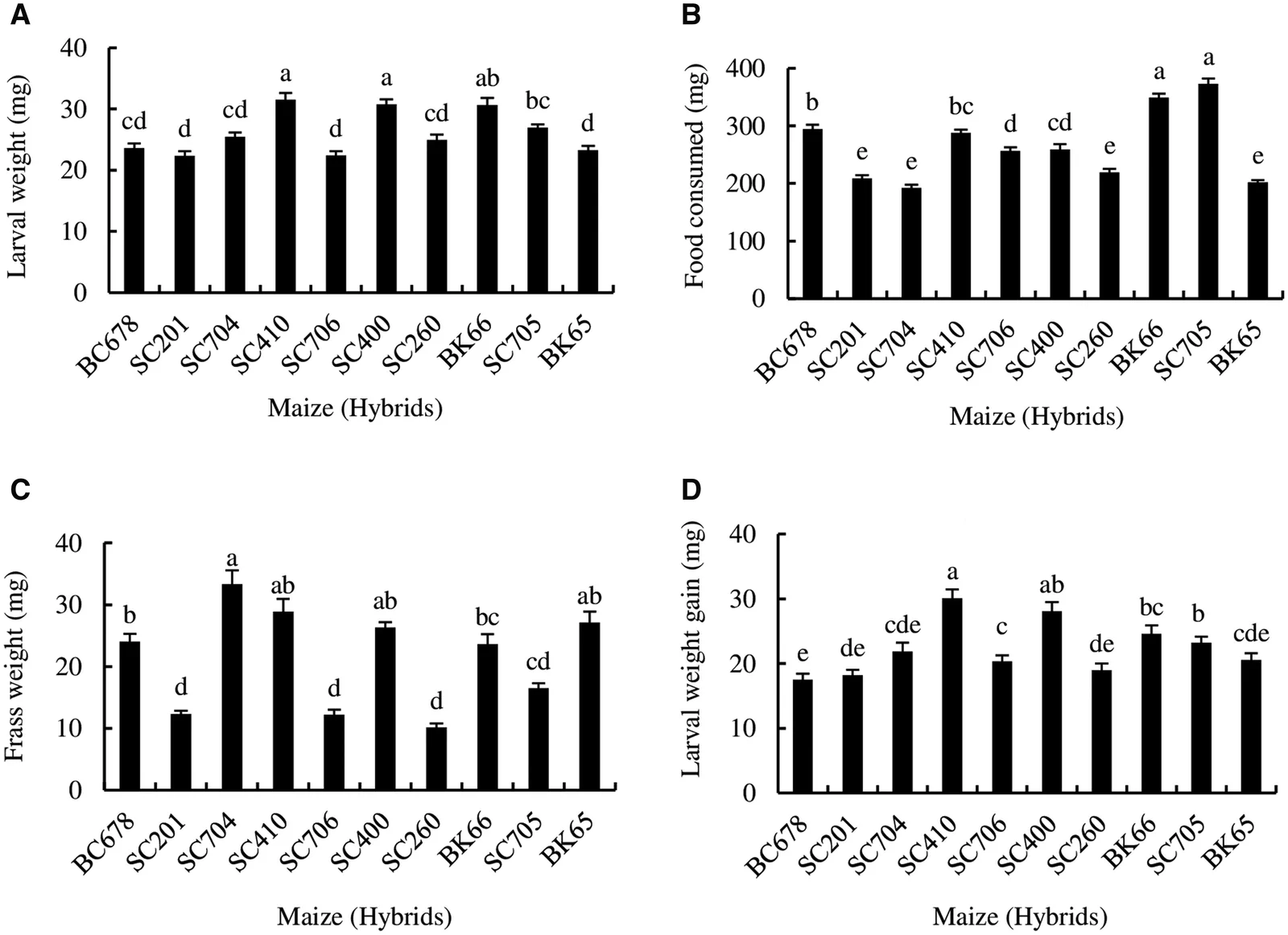
Larval weight A), food consumed B), frass weight C), and larval weight gain D) (mean ± SE) of *Spodoptera frugiperda* larvae fed on the kernels of 10 maize hybrids. Columns labeled with different letters are significantly different (Tukey’s HSD test, *P *< 0.05).

**Fig. 2.**
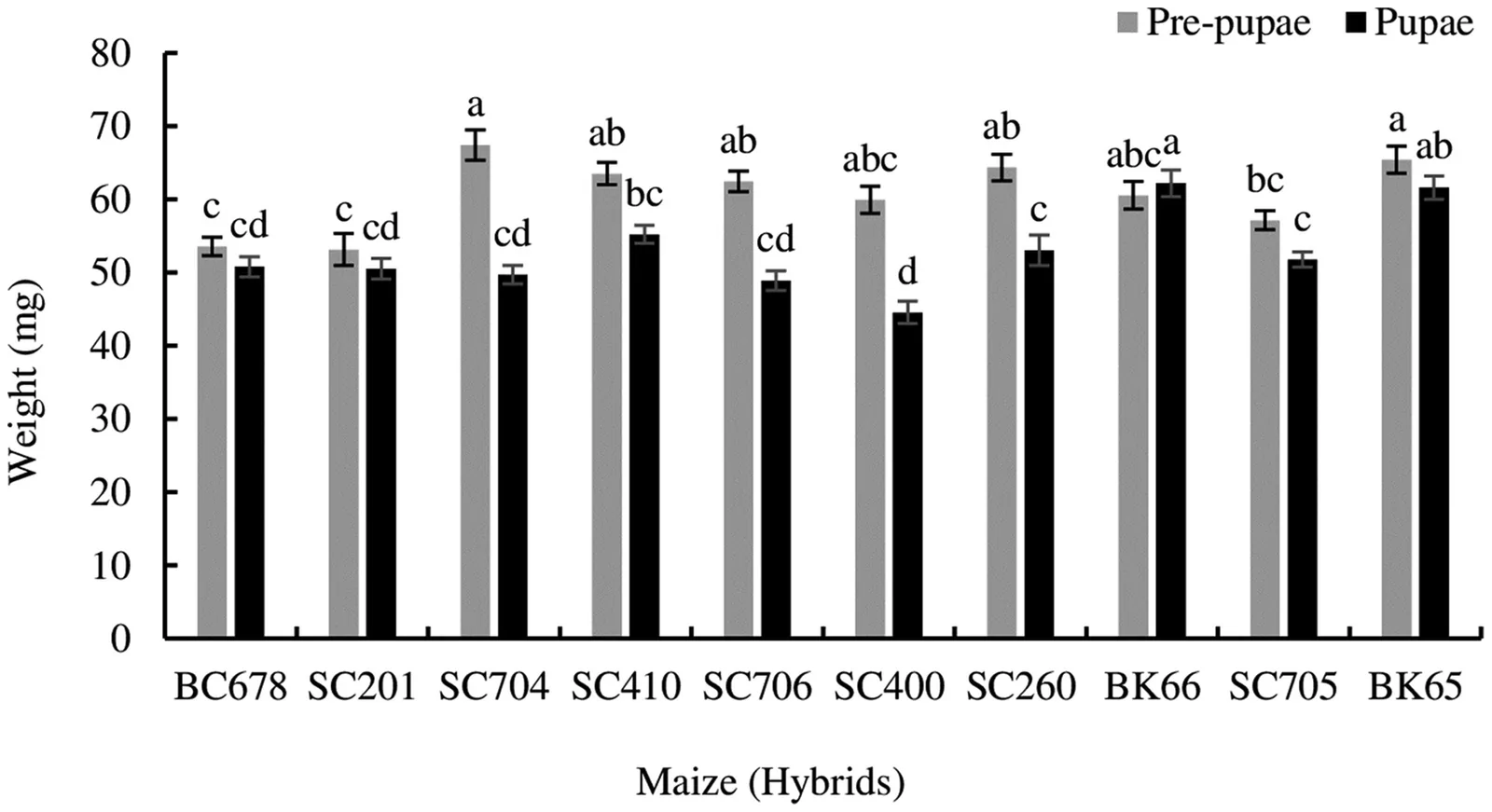
Prepupal and pupal weights (mean ± SE) of *Spodoptera frugiperda* reared on the kernels of 10 maize hybrids. Columns labeled with different letters are significantly different (Tukey’s HSD test, *P *< 0.05).

Significant hybrid-dependent differences were found in larval weight, food consumed, frass weight, larval weight gain, and prepupal and pupal weights (Figs 1A–D and 2). Larvae reared on SC201, SC706, and BK65 had lower weights, whereas those fed on SC410 and SC400 showed greater weights (*F *= 19.08; df = 9, 240; *P *< 0.001) (Fig. 1A). Food consumed was low on SC201, SC704, SC260, and BK65, while it was enhanced on SC705 and BK66 (*F *= 84.09; df = 9, 240; *P *< 0.001) (Fig. 1B). The greatest frass weight was obtained on SC704, while the lowest weights were documented on SC201, SC706, and SC260 (*F *= 24.24; df = 9, 240; *P *< 0.001) (Fig. 1C). Weight gain was lowest on BC678, whereas its was highest on SC410 (*F *= 13.32; df = 9, 240; *P *< 0.001) (Fig. 1D). Prepupal weight was decreased in individuals reared on BC678 and SC201, but it was peaked in those maintained on SC704 and BK65 (*F *= 7.83; df = 9, 240; *P *< 0.001) (Fig. 2). The lowest and highest pupal weights were recorded on SC400 and BK66 (*F *= 13.73; df = 9, 240; *P *< 0.001) (Fig. 2).

### Demographic Parameters of *Spodoptera frugiperda*

The developmental duration and survival of immature stages of *S. frugiperda* on the kernels of various maize hybrids are presented in Table 2. The mean development time reduced significantly when *S. frugiperda* reared on BK65, while it was extended rearing on SC201 (*P *< 0.05). The egg incubation period and prepupal period were statistically the same for all tested maize hybrids (*P *> 0.05). The larvae completed its development significantly longer on BC678 and SC705, but shorter on SC410 and SC706 (*P *< 0.05). The highest and lowest pupal periods were on SC410 and BK65, respectively (*P *< 0.05). Individuals reared on SC400, SC260, BK66, and BK65 showed reduced preadult survival, whereas those reared on SC705 exhibited increased survival (*P *< 0.05).

**Table 2.** Developmental duration and survival (mean ± SE) of *Spodoptera frugiperda* reared on the kernels of 10 maize hybrids

Hybrid	Development time (day)	Egg incubation (day)	Larval period (day)	Prepupal period (day)	Pupal period (day)	Preadult survival
**BC678**	26.9 ± 0.3ab	2.0 ± 0.01a	11.9 ± 0.1a	2.0 ± 0.01a	11.1 ± 0.2c	0.64 ± 0.07bc
**SC201**	27.3 ± 0.2a	2.0 ± 0.03a	11.5 ± 0.1 b	2.0 ± 0.03a	11.8 ± 0.1ab	0.64 ± 0.07bc
**SC704**	25.6 ± 0.2c	2.0 ± 0.03a	11.0 ± 0.1c	2.0 ± 0.02a	10.5 ± 0.2d	0.72 ± 0.06abc
**SC410**	25.7 ± 0.1c	2.0 ± 0.01a	9.8 ± 0.1e	2.0 ± 0.01a	11.8 ± 0.1a	0.80 ± 0.06ab
**SC706**	25.4 ± 0.1c	2.0 ± 0.02a	9.8 ± 0.1e	2.5 ± 0.03a	11.1 ± 0.4 cd	0.78 ± 0.06ab
**SC400**	25.5 ± 0.4c	2.0 ± 0.03a	10.5 ± 0.2d	2.0 ± 0.02a	11.1 ± 0.2c	0.54 ± 0.07c
**SC260**	26.6 ± 0.2 b	2.0 ± 0.02a	10.8 ± 0.1d	2.0 ± 0.01a	11.6 ± 0.1 b	0.58 ± 0.07c
**BK66**	27.0 ± 0.2ab	2.0 ± 0.03a	11.7 ± 0.1ab	2.0 ± 0.01a	11.3 ± 0.1c	0.60 ± 0.07c
**SC705**	26.3 ± 0.3 b	2.0 ± 0.03a	11.8 ± 0.1a	2.0 ± 0.02a	10.4 ± 0.2d	0.84 ± 0.05a
**BK65**	23.9 ± 0.2d	2.0 ± 0.01a	10.6 ± 0.1d	2.0 ± 0.01a	9.3 ± 0.2e	0.60 ± 0.07c

Means followed by different letters in each column are significantly different (paired bootstrap test, *P *< 0.05).

Females reared on SC400 exhibited a longer APOP after eclosion than those reared on SC704 (*P *< 0.05). The TPOP was significantly longer for females reared on BC678 and SC400 compared to those on SC704 (*P *< 0.05). The shortest oviposition period was observed when *S. frugiperda* reared on SC400 (*P *< 0.05). The average of fecundity varied from 201.09 egg/female on SC201 to 663.06 egg/female on BK66 (*P *< 0.05). Female adults lived significantly shorter when reared on SC201, SC704, SC410, and SC260, and longer when fed on BK66 (*P *< 0.05). The longevity of males was significantly shortest on SC260 and longest on SC704 (*P *< 0.05) (Table 3).

**Table 3.** Adult preoviposition period (APOP), total preoviposition period (TPOP), oviposition period, fecundity, and longevity (mean ± SE) of *Spodoptera frugiperda* reared on the kernels of 10 maize hybrids

Hybrid	APOP (day)	TPOP (day)	Oviposition period (day)	Fecundity (offspring)	Female adult longevity (day)	Male adult longevity (day)
**BC678**	7.2 ± 0.6abc	33.4 ± 0.7a	3.4 ± 0.3a	487.6 ± 84.8abc	14.2 ± 0.6 b	11.9 ± 0.9bc
**SC201**	6.4 ± 0.6bcd	32.9 ± 0.9ab	3.4 ± 0.3a	201.1 ± 48.3d	11.6 ± 1.2c	12.0 ± 0.6bc
**SC704**	4.0 ± 0.4e	28.3 ± 0.5e	4.3 ± 0.6a	524.3 ± 137.5abc	11.7 ± 1.0c	15.4 ± 0.6a
**SC410**	6.2 ± 0.4 cd	31.8 ± 0.5abcd	3.0 ± 0.3a	404.8 ± 69.4bc	12.0 ± 0.7c	12.2 ± 0.8bc
**SC706**	5.5 ± 0.5 cd	30.5 ± 0.7 cd	4.3 ± 0.5a	545.7 ± 96.4ab	13.1 ± 0.8bc	13.6 ± 0.5 b
**SC400**	9.0 ± 1.1a	33.4 ± 0.8a	2.0 ± 0.2 b	327.7 ± 60.6bc	14.6 ± 0.8ab	14.7 ± 1.3ab
**SC260**	5.0 ± 0.6de	32.0 ± 0.3abc	5.5 ± 1.5a	403.5 ± 140.7bcd	12.1 ± 0.6c	9.4 ± 1.5c
**BK66**	5.3 ± 0.4d	31.4 ± 0.3bcd	4.9 ± 0.3a	663.1 ± 89.2a	15.8 ± 0.4a	14.5 ± 0.8ab
**SC705**	7.2 ± 0.7abc	32.0 ± 0.0ab	2.7 ± 0.5ab	251.8 ± 66.2 cd	12.4 ± 1.4bc	13.8 ± 0.5 b
**BK65**	7.6 ± 0.6ab	30.3 ± 0.7d	2.9 ± 0.5ab	367.9 ± 103.5bc	12.9 ± 1.2bc	13.1 ± 1.1bc

Means followed by different letters in each column are significantly different (paired bootstrap test, *P *< 0.05).

**Fig. 3.**
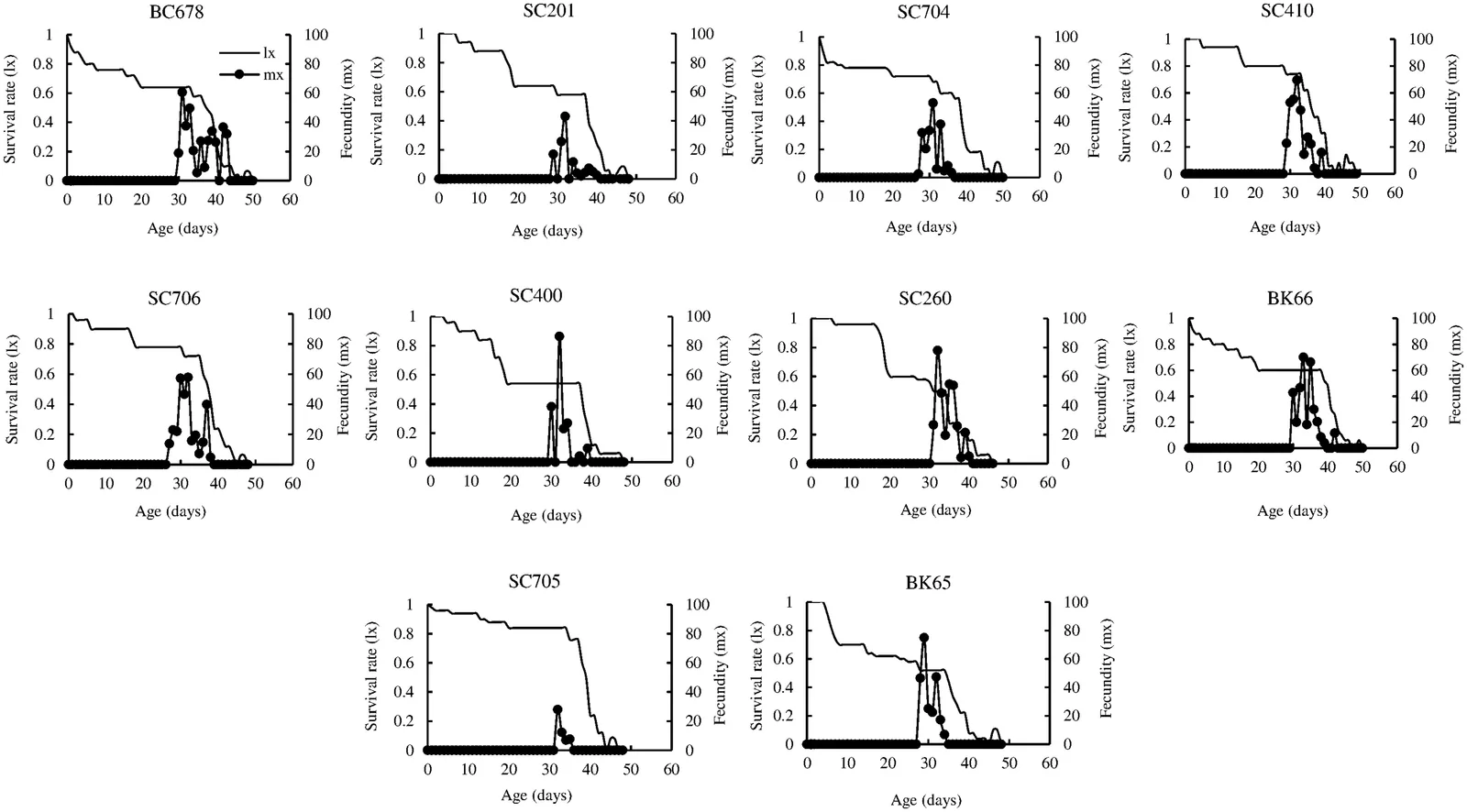
Age-specific survival rate (*l_x_*) and age-specific fecundity (*m_x_*) of *Spodoptera frugiperda* reared on the kernels of 10 maize hybrids.

The age-specific survival rate (*l_x_*) had a decreasing trend as *S. frugiperda* aged on the studied maize hybrids (Fig. 3). The age-specific fecundity (*m_x_*) curves also revealed that *S. frugiperda* produced less offspring on SC705 and SC201 (Fig. 3).

The studied maize hybrids significantly affected the life table parameters of *S. frugiperda* (*P *< 0.05), with results presented in Table 4. The intrinsic rate of increase (*r*) was observed to range between 0.110 and 0.169 day^−1^, with the lower value recorded on SC705 and the higher values obtained on SC410 and SC706. The finite rate of increase (*λ*) showed a pattern similar to that of *r*. The gross reproductive rate (*GRR*) on SC705 and BC678 were 54.69 and 387.18 offspring, respectively, with the latter being more than 7 times the former value. The minimum values of net reproductive rate (*R*_0_) were 45.33 and 68.63 offspring when reared on SC705 and SC201, while its maximum value was 228.75 on SC706. The mean generation time (*T*) was shorter on SC704 and BK65, but longer on BC678, SC260, BK66, and SC705.

**Table 4.** Intrinsic rate of increase (*r*), finite rate of increase (*λ*), gross reproductive rate (*GRR*), net reproductive rate (*R*_0_), and mean generation time (*T*) (mean ± SE) of *Spodoptera frugiperda* reared on the kernels of 10 maize hybrids

Hybrid	*r* (day^−1^)	*𝜆* (day^−1^)	*GRR* (offspring)	*R*_0_ (offspring)	*T* (day)
**BC678**	0.15 ± 0.01ab	1.16 ± 0.01ab	387.2 ± 86.7a	185.3 ± 46.1abc	34.5 ± 0.6a
**SC201**	0.13 ± 0.01bc	1.13 ± 0.01bc	123.2 ± 32.9 cd	68.6 ± 21.1d	33.1 ± 0.5 b
**SC704**	0.15 ± 0.01ab	1.16 ± 0.01ab	202.4 ± 66.9abc	136.8 ± 47.5bcd	31.4 ± 0.3c
**SC410**	0.16 ± 0.01a	1.18 ± 0.01a	331.7 ± 60.8abc	226.8 ± 47.9ab	32.8 ± 0.3bc
**SC706**	0.17 ± 0.01a	1.18 ± 0.01a	322.2 ± 73.4abc	228.7 ± 55.4a	33.0 ± 99.6bc
**SC400**	0.14 ± 0.01bc	1.14 ± 0.01bc	187.4 ± 46.7bc	98.3 ± 27.7 cd	33.1 ± 0.3 b
**SC260**	0.14 ± 0.01abc	1.15 ± 0.01abc	338.7 ± 117.5abc	128.8 ± 51.3bcd	34.3 ± 0.3a
**BK66**	0.15 ± 0.01ab	1.16 ± 0.01ab	339.5 ± 76.2ab	199.9 ± 50.5abc	34.2 ± 0.4a
**SC705**	0.11 ± 0.01c	1.12 ± 0.01c	54.7 ± 21.1d	45.3 ± 17.7d	33.8 ± 0.1a
**BK65**	0.15 ± 0.01ab	1.16 ± 0.01ab	240.6 ± 75.7abc	125.1 ± 42.6bcd	30.9 ± 0.3c

Means followed by different letters in each column are significantly different (paired bootstrap test, *P *< 0.05).

### Digestive Enzymes of *Spodoptera frugiperda*

Protease activity was significantly highest on the kernels of SC706, whereas its activity was decreased on SC704, BK66, SC705, and BK65 (*F *= 11.44; df = 9, 20; *P *< 0.001) (Table 5). The larvae reared on SC410 and BK65 significantly exhibited higher activity of amylase, while those fed on SC705 showed minimal enzyme activity in their midguts (*F *= 3.27; df = 9, 20; *P *= 0.01) (Table 5).

**Table 5.** General proteolytic and amylolytic activities (mean ± SE) in *Spodoptera frugiperda* larvae fed on the kernels of 10 maize hybrids

Hybrids	Protease (U mg^−1^)	Amylase (U mg^−1^)
**BC678**	0.5 ± 0.0ab	3.1 ± 0.1ab
**SC201**	0.4 ± 0.0bc	3.3 ± 0.0ab
**SC704**	0.1 ± 0.0c	4.0 ± 0.1ab
**SC410**	0.3 ± 0.0bc	4.5 ± 0.1a
**SC706**	0.8 ± 0.0a	3.7 ± 0.0ab
**SC400**	0.4 ± 0.0bc	1.6 ± 0.0ab
**SC260**	0.3 ± 0.0bc	3.6 ± 0.0ab
**BK66**	0.1 ± 0.0c	3.6 ± 0.0ab
**SC705**	0.1 ± 0.0c	2.3 ± 0.1 b
**BK65**	0.1 ± 0.0c	4.4 ± 0.0a

Means followed by different letters in each column are significantly different (Tukey’s HSD test, *P *< 0.05).

### Antioxidant Enzymes of *Spodoptera frugiperda*

In this study, the highest POD activity was found in larvae reared on the kernels of SC705, while lower activities of POD were occurred in insects fed on SC260 and BK65 (*F *= 47.91; df = 9, 20; *P *< 0.001) (Table 6). *Spodoptera frugiperda* larvae fed on BC678 and SC705 had significantly higher levels of SOD activities (*F *= 18.19; df = 9, 20; *P *< 0.001). However, lower activities of the enzyme were recorded in larvae reared on SC410, SC706, and SC400 (Table 6). Additionally, larvae fed on BK65 showed the highest CAT activity, but those fed on SC706 and BK66 exhibited lower activities (*F *= 13.59; df = 9, 20; *P *< 0.001) (Table 6).

**Table 6.** Antioxidant enzymes activities (mean ± SE) in *Spodoptera frugiperda* larvae fed on the kernels of 10 maize hybrids

Hybrids	POD (U mg^−1^)	SOD (U mg^−1^)	CAT (U mg^−1^)
**BC678**	0.17 ± 0.01bc	62.6 ± 5.3a	0.07 ± 0.01ab
**SC201**	0.11 ± 0.01c	36.6 ± 3.1bcd	0.05 ± 0.02bcd
**SC704**	0.07 ± 0.01 cd	29.8 ± 3.1 cd	0.01 ± 0.00 cd
**SC410**	0.12 ± 0.01bcd	23.8 ± 2.2d	0.07 ± 0.01 b
**SC706**	0.24 ± 0.02 b	19.8 ± 1.5d	0.01 ± 0.00d
**SC400**	0.13 ± 0.02bcd	24.9 ± 2.1d	0.04 ± 0.00bcd
**SC260**	0.04 ± 0.00d	48.9 ± 4.2ab	0.06 ± 0.01bc
**BK66**	0.12 ± 0.02 cd	49.3 ± 4.2ab	0.01 ± 0.00d
**SC705**	0.58 ± 0.06a	62.6 ± 5.2a	0.08 ± 0.01ab
**BK65**	0.04 ± 0.00d	45.0 ± 3.7abc	0.12 ± 0.02a

Means followed by different letters in each column are significantly different (Tukey’s HSD test, *P *< 0.05).

Abbreviations: CAT, catalase; POD, peroxidase; SOD, superoxide dismutase.

### Primary and Secondary Metabolites in Maize Hybrids Kernels

Statistically significant differences were found in primary and secondary metabolite levels in the kernels (*P *< 0.05) (Table 7). The results indicated that SC704, SC410, SC706, SC400, and BK66 had higher carbohydrate contents in their kernels compared to other hybrids (*F *= 41.34; df = 9, 20; *P *< 0.001). Increased starch levels were also observed in SC704, SC410, SC706, and BK66, while the level of this primary metabolite was low in other hybrids (*F *= 31.09; df = 9, 20; *P *< 0.001). Kernels of SC201 and SC705 had higher protein contents, whereas those of SC705 and BK65 contained lower levels (*F *= 5.23; df = 9, 20; *P *< 0.001). Hybrid SC705 accumulated the highest content of total phenol in its kernels, whereas kernels of SC410 and BK66 had lower amounts of this secondary metabolite (*F *= 9.28; df = 9, 20; *P *< 0.001). Flavonoid content in the kernels of BC678 was maximum, while it was minimum in SC201 and SC410 (*F *= 3.63; df = 9, 20; *P *< 0.001). Moreover, the highest concentration of anthocyanin was detected in the kernels of SC704 (*F *= 4.74; df = 9, 20; *P *< 0.001).

**Table 7.** Primary and secondary metabolites (mean ± SE) quantified from the kernels of 10 maize hybrids

Hybrids	Carbohydrate (%)	Starch (%)	Protein (%)	Total phenol (mg g^−1^)	Flavonoid (mg g^−1^)	Anthocyanin (mg g^−1^)
**BC678**	2.2 ± 0.2 b	0.8 ± 0.1 b	9.2 ± 0.4ab	2.9 ± 0.1abcd	16.4 ± 1.4a	0.019 ± 0.002ab
**SC201**	1.7 ± 0.1 b	0.8 ± 0.1 b	11.9 ± 0.4a	2.7 ± 0.1bcd	9.9 ± 0.8 b	0.016 ± 0.001 b
**SC704**	13.4 ± 1.3a	5.6 ± 0.8a	9.3 ± 0.4ab	2.9 ± 0.2abcd	14.2 ± 1.2ab	0.052 ± 0.002a
**SC410**	10.5 ± 0.9a	6.6 ± 0.5a	9.3 ± 0.8ab	2.1 ± 0.1d	9.7 ± 0.8 b	0.009 ± 0.001 b
**SC706**	9.9 ± 0.8a	6.4 ± 0.5a	11.7 ± 0.7a	3.1 ± 0.1abc	14.1 ± 1.2ab	0.011 ± 0.001 b
**SC400**	12.6 ± 1.1a	5.4 ± 0.5 b	9.7 ± 0.8ab	3.5 ± 0.2ab	13.3 ± 1.1ab	0.007 ± 0.001 b
**SC260**	2.1 ± 0.2 b	0.9 ± 0.1 b	9.0 ± 0.9ab	2.3 ± 0.2 cd	11.3 ± 1.0ab	0.005 ± 0.001 b
**BK66**	9.8 ± 1.1a	4.4 ± 0.8a	10.2 ± 0.3ab	2.2 ± 0.2d	14.2 ± 2.2ab	0.020 ± 0.001ab
**SC705**	1.4 ± 0.1 b	1.0 ± 0.1 b	7.2 ± 0.2 b	3.8 ± 0.1a	14.7 ± 1.3ab	0.009 ± 0.001 b
**BK65**	2.6 ± 0.2 b	0.7 ± 0.1 b	8.4 ± 0.7 b	2.6 ± 0.2bcd	11.6 ± 1.3ab	0.031 ± 0.003ab

Means followed by different letters in each column are significantly different (Tukey’s HSD test, *P *< 0.05).

### Correlation Analysis

**Fig. 4.**
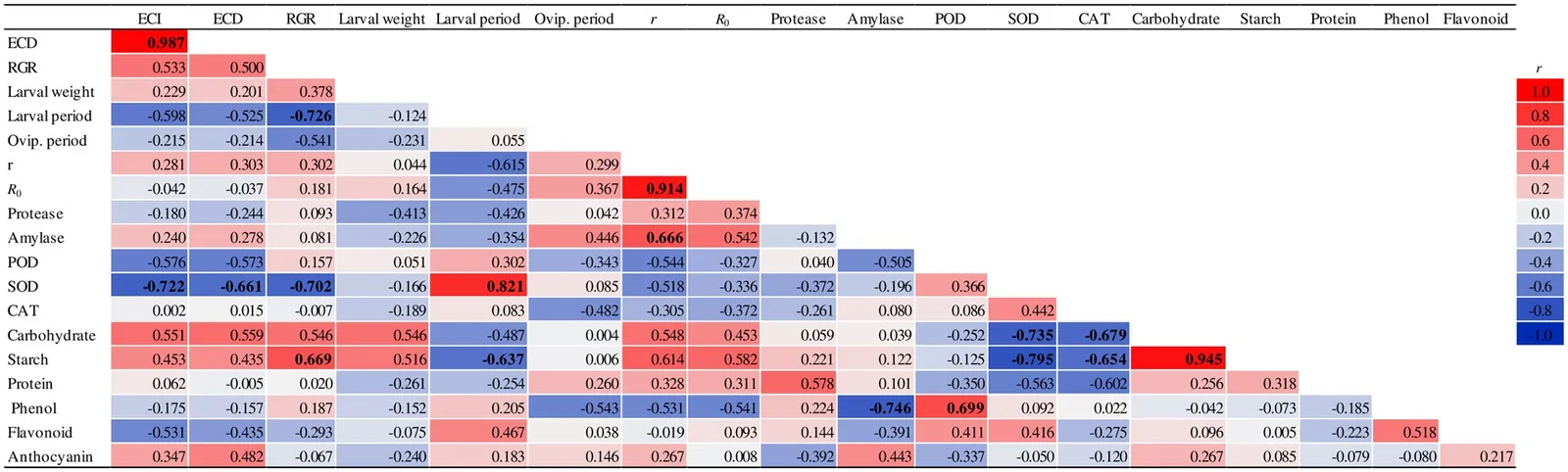
Matrix of Pearson’s correlation coefficients among nutritional indices, demographic performance, and enzymatic activities of *Spodoptera frugiperda* and primary and secondary metabolites of maize hybrids kernels. Bold numbers indicate statistically significant correlations (*P *< 0.05). Color intensity reflects correlation strength: red indicates stronger positive correlations, while darker blue shows stronger negative correlations. Abbreviations: ECD, efficiency of conversion of digested food; ECI, efficiency of conversion of ingested food; RGR, relative growth rate; Ovip. Period, oviposition period; *r*, intrinsic rate of increase; *R*_0_, net reproduction rate; POD, peroxidase; SOD, superoxide dismutase; CAT, catalase.

As shown in Fig. 4, ECI exhibited a direct relationship with ECD (*r *= 0.986; df = 8; *P *< 0.001) and an inverse relationship with SOD activity (*r *= −0.721; df = 8; *P *= 0.01). Likewise, ECD displayed a significant negative association with SOD activity (*r *= 0 − 0.661; df = 8; *P *= 0.03). The RGR was correlated negatively with larval period (*r *= −0.725; df = 8; *P *= 0.01) and SOD activity (*r *= −0.702; df = 8; *P *= 0.02). The larval period exhibited a significant positive correlation with SOD activity (*r *= 0.821; df = 8; *P *< 0.01) and a negative one with starch content (*r *= −0.637; df = 8; *P *= 0.04). Significant positive correlations were also found between *r* and *R*_0_ (*r *= 0.913; df = 8; *P *< 0.001) as well as amylase activity (*r *= 0.665; df = 8; *P *= 0.03). A significant negative association was detected between amylase activity and phenol content (*r *= −0.746; df = 8; *P *= 0.01). A positive relationship was detected between POD activity and phenol content (*r *= 0.699; df = 8; *P *= 0.02). The SOD activity had negative relationships with carbohydrate (*r *= −0.734; df = 8; *P *= 0.01) and starch (*r *= −0.795; df = 8; *P *< 0.01) contents. The same relationships were observed for CAT activity (carbohydrate: *r *= −0.678; df = 8; *P *= 0.03) and (starch: *r *= −0.654; df = 8; *P *= 0.04). Finally, carbohydrate content was positively correlated with starch content (*r *= 0.945; df = 8; *P *< 0.001).

### Cluster Analysis

**Fig. 5.**
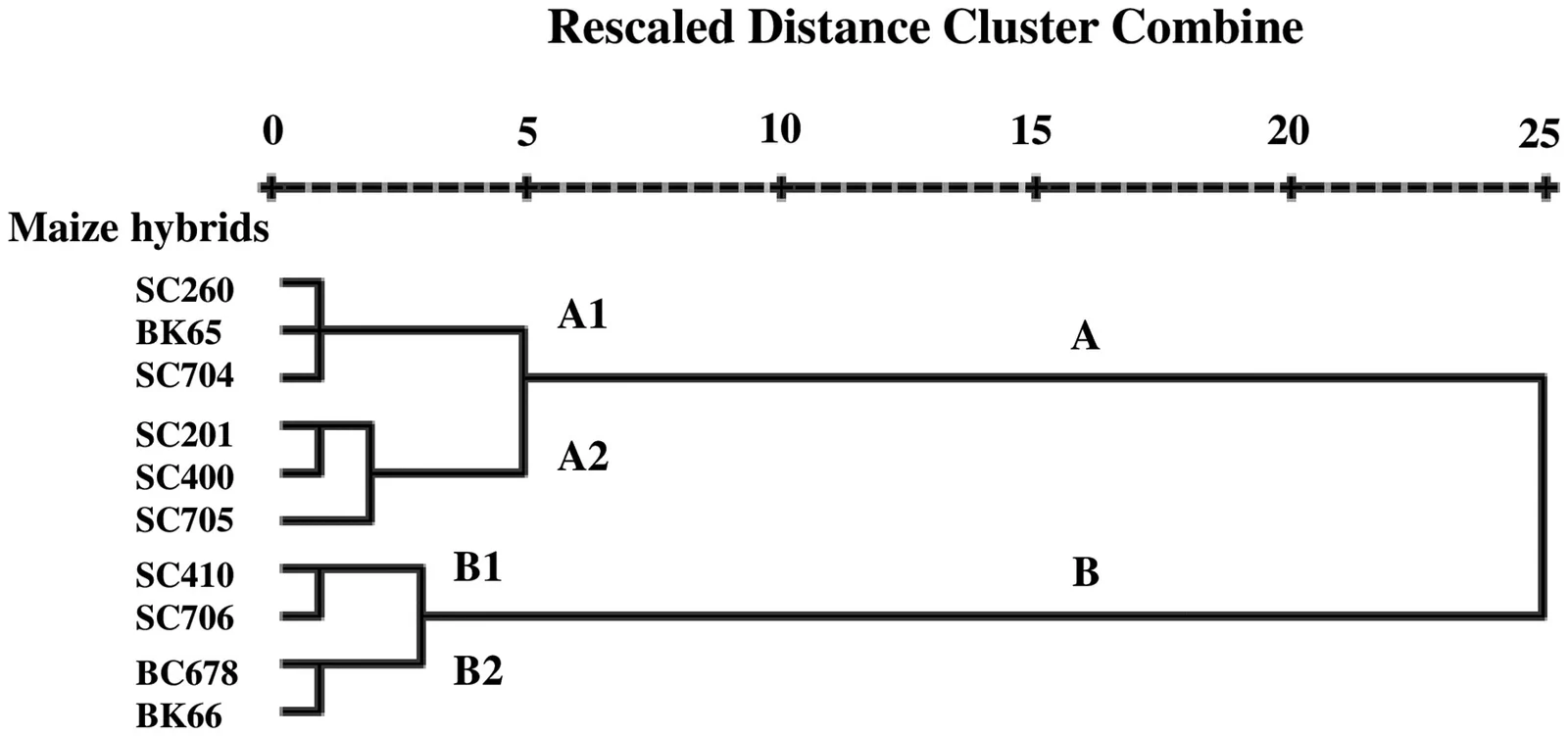
Dendrogram of maize hybrids based on nutritional indices, demographic performance, and enzymatic activities of *Spodoptera frugiperda* fed on the kernels of 10 maize hybrids, constructed using Ward’s minimum-variance method.

According to the hierarchical clustering results (Fig. 5), the nutritional indices, demographic features, and enzyme responses of *S. frugiperda* fed on the kernels of 10 maize hybrids formed 2 chief clusters, designated A and B. This grouping was supported by the agglomeration schedule, which showed a pronounced increase in the agglomeration coefficient at the final clustering stage (from 10,557.22 to 39,466.13), indicating the presence of 2 distinct hybrid clusters, as illustrated in the dendrogram (Fig. 5). Within cluster A, 2 subclusters emerged: A1, which included SC260, BK65, and SC704 hybrids, and A2, which comprised hybrids SC201, SC400, and SC705, distributed across 2 separate groups. Cluster B was also subdivided, with B1 containing the SC410 and SC706 hybrids, and B2 comprising of BC678 and BK66 hybrids.

## Discussion

Host plant quality influences the nutritional, biological, and physiological traits of herbivorous insects (Shishehbor and Hemmati 2022, Hosseini Mousavi et al. 2023, Jafari et al. 2025a, 2025b). In the current study, we applied a stepwise framework connecting *S. frugiperda* performance traits with the biochemical properties of various maize hybrids to provide a mechanistic explanation for the observed variation in plant–pest interactions.

Nutritional indices differed significantly among maize hybrids. Larvae fed on SC705 showed higher food consumption, CI, RCR, and AD, but lower ECI and ECDfood. Similarly, larvae feeding on BC678 also exhibited reduced ECI and ECD. These findings indicate that although the kernels of these hybrids were readily consumed and digested, the assimilated nutrients were converted into biomass less efficiently. The ECI and ECD indicates the efficiency of converting ingested and digested food into body tissue, respectively (Nathan et al. 2006, Pinto et al. 2019). Reduced biomass accumulation is frequently associated with prolonged larval development and reduced adult fecundity (Shishehbor and Hemmati 2022).

RMR reflects energy expenditure on maintenance metabolism, whereas MC represents the energetic cost of metabolism (Pinto et al. 2019). The elevated RMR and MC observed on SC705 and BC678 indicate greater energy allocation to maintenance rather than growth and reproduction, likely due to nutrient imbalances or antinutritional compounds (Jafari et al. 2025a). Consistent with this, the kernels of SC705 and BC678 contained low levels of carbohydrates, starch, and protein but high concentrations of total phenols and flavonoids, which may have reduced the larvae efficiency in converting nutrients into biomass. Phenolic compounds, such as flavonoids, in the kernel pericarp are thought to function both as a physical barrier against chewing pests, by strengthening the pericarp structure, and as a biochemical deterrent, as dense cell wall compositions and flavonoid-rich extracts (e.g. polyphenols) can reduce insect feeding activity and delay larval development (Venturini et al. 2016, Tayal et al. 2020). However, further studies employing liquid chromatography-mass spectrometry or related metabolomic techniques are needed to characterize the actual bioactive compounds responsible for reduced efficiency of *S. frugiperda* and clarify their individual contributions to maize resistance. Previous studies have shown that high levels of plant secondary metabolites and ROS-induced oxidative products can damage the larval midgut, reducing biomass accumulation (Appel and Martin 1992, Ramalho et al. 2011, Shishehbor and Hemmati 2022). Reduced ECI and ECD were documented by Alekaram et al. (2024) for *Spodoptera littoralis* (Boisd) fed on a maize hybrid (Oteel) seed-based diet. Jafari et al. (2025a) also reported lower ECI and ECD and higher MC in *L. loreyi* larvae fed on the leaves of SC706. The contrasting hybrid responses between these studies and the present work likely reflect differences in insect species, plant tissues, nutritional and defensive traits, agronomic practices, and environmental conditions.

In contrast, lower consumption (CR and RCR), digestibility (AD), and metabolic rate (RMR) were observed on SC704. Moreover, the low metabolic cost (MC) on SC704 and SC400 suggests reduced energy expenditure on digestion, absorption, and nutrient processing (Pinto et al. 2019), allowing more energy to be allocated to growth and reproduction, as reflected by higher ECI and ECD. The high carbohydrate and starch contents likely enhanced the nutritional quality of SC704 and SC400 for the pest. Furthermore, larval weight, weight gain, and RGR, which reflects daily insect biomass accumulation relative to body weight (Pinto et al. 2019, Truzi et al. 2021), were highest on SC410. These findings indicate that this hybrid provided a high-quality nutritional source, likely due to its higher carbohydrate and starch contents and lower levels of defensive secondary metabolites, thereby promoting faster population growth. Correlation analysis further showed a positive relationship between RGR and starch content, consistent with previous studies linking increased RGR to improved host food quality (Truzi et al. 2021, Jafari et al. 2025b). Higher RGR was similarly reported by Alekaram et al. (2024) and Jafari et al. (2025a) for *S. littoralis* larvae reared on SC703 seed-based artificial diet and *L. loreyi* larvae fed on BC678 leaves, respectively. The varied responses among maize hybrids are likely attributable to differences in insect species and the plant hybrids and tissues evaluated.

Demographic analysis of *S. frugiperda*, based on life table parameters and life history traits, demonstrated significant variations among the maize hybrids. Insects reared on SC201 exhibited prolonged development time, accompanied by lower fecundity and reduced adult female longevity. Slower development and lower reproductive output of insect pests on a specific host plant often indicate reduced host suitability and the presence of plant resistance traits (Chen et al. 2018). Similarly, Zhang et al. (2023) reported that S*. frugiperda* adopted a compensatory strategy by increasing larval duration when reared on a less suitable maize cultivar (Zhengdan 958). Lower carbohydrate and starch contents of SC201 likely limited energy availability for larval growth and reproduction, resulting in prolonged development and reduced fecundity (van Schoor et al. 2020). Because life table parameters integrate the cumulative impacts of host plants on insect survival, development, and reproduction throughout the life cycle, they provide a more comprehensive assessment than nutritional indices or enzymatic activities measured at specific developmental stages (Bonvari et al. 2024).

Findings from the life table experiment indicated that *S. frugiperda* reared on SC705 kernels exhibited a lower intrinsic rate of increase (*r*), finite rate of increase (*λ*), net reproduction rate (*R*_0_), and *GRR*. Conversely, the mean generation time (*T*) and larval duration were significantly higher on SC705, suggesting that this hybrid exhibits a resistant reaction to the pest. The suppressed demographic parameters observed on SC705 kernels, particularly the intrinsic rate of increase (*r*), a key determinant of host plant suitability (Xie et al. 2021, Zhou et al. 2022), are likely related to the reduced concentrations of nutritional compounds (carbohydrate, starch, and protein) and elevated levels of total phenol. Similarly, Zhang et al. (2023) reported suppressed *S. frugiperda* population growth on a resistant maize variety (Zhengdan 958), as indicated by lower *r*, *λ*, *R*_0_, values, alongside a longer *T*. In contrast, Lu et al. (2024) reported *r* and *R*_0_ values of 0.1336 day^−1^ and 135.5 offspring, respectively, for *S. frugiperda* reared on leaves of the resistant silage maize cultivar Tongli 8. The corresponding values in the present study were lower. In addition to inherent differences among maize cultivars, this variation may reflect the plant tissues used for insect rearing. Lu et al. (2024) used leaves, the preferred feeding site of *S. frugiperda*, whereas kernels were used in the present study. These tissues differ markedly in biochemical composition, and even within leaves, differences in epidermal lipid composition can significantly influence *S. frugiperda* feeding and development (Yang et al. 1993).

In contrast, insects reared on SC410 and SC706 exhibited shorter larval durations and prolonged oviposition periods, alongside higher of *r*, *λ*, and *R*_0_ values. These findings suggest that SC410 and SC706 provided more favorable nutritional conditions for *S. frugiperda* development, reproduction, and population growth. This is likely due to their higher levels of carbohydrates, starch, and protein, and lower concentrations of phenolics, flavonoids, and anthocyanins. Similar studies have shown that carbohydrate and protein contents are key determinants of insect development and population growth and vary among host plant species and cultivars (Cabezas et al. 2013, da Silva et al. 2017, Truzi et al. 2021, Jafari et al. 2025b).

Larvae of *S. frugiperda* fed on SC705 exhibited reduced protease and amylase activities, likely due to the lower protein, carbohydrate, and starch contents of its kernels. These reductions may have contributed to the lower ECI and ECD values, prolonged larval development, and reduced population growth. In contrast, the higher amylase and protease activities observed on SC410 and SC706, respectively, were likely associated with their greater nutritional contents, indicating an enhanced digestive response to support larval growth (Pyati et al. 2011). In accordance with the present results, Alekaram et al. (2024), Arab Yabarati et al. (2025), and Jafari et al. (2025b) reported that variations in the concentrations of nutritional compounds among different hybrids or cultivars significantly influence the activities of digestive enzymes in lepidopteran larvae. Moreover, the increased activities of POD and SOD in *S. frugiperda* larvae fed on SC705 suggest an enhanced antioxidant defense response that may be associated with high phenolic content and other defensive secondary metabolites in the kernels. Correlation analysis further confirmed a significant positive association between POD activity and total phenol concentration. Elevated activities of antioxidant enzymes reflect increased investment in antioxidant defense (Deska 2020). Such physiological responses may increase metabolic costs and potentially affect energy metabolism and nutrient utilization (Mardani-Talaee et al. 2025), thereby explaining the reduced larval performance on SC705. This finding aligns with earlier report by Jafari et al. (2025b), which linked higher concentrations of plant secondary metabolites to enhanced antioxidant defense enzyme activities in herbivorous insects.

Overall, the present study demonstrates that the kernels of different maize hybrids vary considerably in their suitability for *S. frugiperda*. The hybrid SC705, which formed a distinct group within subcluster A2 in the hierarchical clustering analysis, exhibited greater resistance to the pest, likely associated with its primary and secondary metabolite profile. In contrast, SC410 and SC706, classified within subcluster B1, exhibited higher nutritional quality and supported superior insect performance. These findings reveal strong associations between kernel biochemical traits and *S. frugiperda* performance, suggesting that these traits may contribute to host plant resistance. Beyond identifying resistant germplasm, the results provide a basis for selecting candidate hybrids for resistance screening and as potential donor materials in breeding programs aimed at improving resistance to *S. frugiperda*. The variation in kernel suitability among maize hybrids identified in the current study may also facilitate the development of standardized screening protocols for evaluating resistance to ear- and kernel-feeding stages of the pest. Hybrid SC705 may also represent a promising candidate for incorporation into integrated pest management programs aimed at minimizing postflowering damage and suppressing *S. frugiperda* population buildup. Future studies should validate its resistance under field conditions and integrate biochemical, genetic, and molecular approaches, including targeted metabolite analyses, to identify the resistance mechanisms and facilitate their incorporation into maize improvement programs.
